# Specialist-mediated play therapy for toddlers with language developmental delay: a single-center observational pilot study

**DOI:** 10.3389/fresc.2026.1830893

**Published:** 2026-05-29

**Authors:** Yingjie Zhan, Rui Liu, John Sieh Dumbuya, Jun Lu

**Affiliations:** 1 Department of Pediatrics, People’s Hospital of Pingshan, Shenzhen, China; 2 Department of Pediatrics, Pingshan Hospital, Southern Medical University, Shenzhen, China; 3 Department of Pediatrics, Inner Mongolia Autonomous Region Maternal and Child Health Hospital, Hohhot, China; 4 Department of Pediatrics, Haikou Affiliated Hospital of Central South University, Xiangya School of Medicine, Central South University, Haikou, China; 5 Department of Pediatric Endocrinology and Well Child Care, Zhujiang Hospital of Southern Medical University, Guangzhou, China

**Keywords:** case–control study, developmental pediatrics, early intervention, language developmental delay, non-randomized, play therapy

## Abstract

**Background:**

Language developmental delay is one of the most common neurodevelopmental concerns in early childhood. Early intervention is considered critical for improving communication outcomes, yet the comparative effectiveness of different intervention approaches remains incompletely understood.

**Objective:**

To examine the association between three early intervention strategies, clinic-based structured language rehabilitation, parent-led family intervention, and specialist-mediated play therapy, and language developmental outcomes in toddlers with mild language developmental delay in a non-randomized observational design.

**Methods:**

This single-center case-control observational pilot study included 180 children aged 18–36 months diagnosed with mild language developmental delay using the Gesell Developmental Diagnosis Scale. No randomization was performed. Participants were categorized into three intervention groups according to the clinical management strategy selected following standardized physician consultation and parental preference: clinic-based structured language rehabilitation (*n* = 60), parent-led family intervention (*n* = 60), and specialist-mediated play therapy (*n* = 60). Language developmental quotient (DQ) scores were assessed at baseline, 6 months, and 12 months. Changes in DQ scores over time were analyzed using repeated-measures analysis of variance.

**Results:**

Language DQ improved significantly over time in all three groups (*p* < 0.001). However, children receiving specialist-mediated play therapy demonstrated greater improvement than those receiving clinic-based structured rehabilitation or parent-led intervention. At 6 months, 43.3% of children in the play therapy group improved to the borderline language category compared with 21.7% in the clinic-based group and 23.3% in the family intervention group. At 12 months, 50.0% of children in the play therapy group achieved language scores within the normal range, compared with 18.3% and 25.0% in the clinic-based and family intervention groups, respectively. A significant group-by-time interaction was observed (*p* < 0.001), suggesting different developmental trajectories across intervention approaches.

**Conclusion:**

Early intervention was associated with improvements in language development among toddlers with mild language delay. Specialist-mediated play therapy demonstrated a stronger association with language gains compared with clinic-based structured rehabilitation and parent-led interventions. These findings demonstrate association rather than causation owing to the non-randomized observational design. Further large-scale, multicenter randomized controlled trials are required to confirm these findings, establish causal efficacy, and determine optimal early intervention strategies.

## 1 Introduction

Language developmental delay (LDD) is one of the most common developmental concerns in early childhood, affecting approximately 5%–10% of preschool-aged children (1, 2). Children with LDD demonstrate significant delays in expressive and/or receptive language compared with age-matched peers. Without timely intervention, early language delays may contribute to long-term difficulties in literacy acquisition, academic performance, social interaction, and emotional well-being (3, 4).

Early intervention programs aim to support language acquisition during critical periods of neurodevelopmental plasticity. Clinic-based structured language rehabilitation approaches for toddlers with LDD often rely on structured, therapist-directed activities designed to target specific linguistic skills, including phonological production, vocabulary development, and sentence construction (5, 6). While such interventions have demonstrated effectiveness, their highly structured format may not always sustain attention or motivation in very young children. Reduced engagement can limit the intensity and generalization of learning within natural social environments (7).

Play represents the primary mode of learning during early childhood and provides a natural context in which communication skills emerge (8). Developmental theories, particularly those derived from social constructivist frameworks, emphasize the importance of interactive learning within meaningful social contexts (9, 10). In play-based environments, children are exposed to rich linguistic input while actively participating in socially meaningful interactions. Such environments facilitate the development of vocabulary, pragmatic language, symbolic representation, and social communication skills (11).

Growing evidence supports the role of naturalistic and play-based intervention approaches in promoting early communication development (12). However, many existing studies focus primarily on therapist-led play sessions or parent-implemented interventions, and relatively few have examined models in which developmental specialists actively design and supervise individualized play-based therapeutic programs (13, 14).

Developmental-behavioral pediatricians possess expertise in neurodevelopmental assessment and early childhood development, allowing them to identify individualized developmental profiles and tailor intervention strategies accordingly. Integrating this diagnostic expertise with structured play-based therapy may enhance the precision and effectiveness of early language intervention.

Therefore, the present study aimed to examine the clinical outcomes associated with a specialist-mediated play therapy model implemented in a pediatric developmental clinic. Specifically, we compared language developmental trajectories among toddlers with mild language delay receiving (1) clinic-based structured language rehabilitation, (2) parent-led family intervention, or (3) specialist-mediated play therapy. As a pilot observational case–control study, the objective was to explore whether the specialist-guided play model is associated with improved language developmental outcomes and to generate preliminary evidence to inform future controlled trials.

## 2 Methods

### 2.1 Study design and participants

This study was conducted as a single-center observational case–control pilot study at the Department of Pediatrics, People's Hospital of Pingshan, Shenzhen, China. The study was carried out between June 2023 and December 2023. A total of 180 children aged 18–36 months who were diagnosed with mild language developmental delay (LDD) were included. The diagnosis was established by a developmental-behavioral pediatrician using the language domain of the Gesell Developmental Diagnosis Scale, which provides a standardized developmental quotient (DQ) for language ability.

No randomization was performed. Participants were categorized into three intervention groups according to the clinical management strategy selected following physician consultation and parental preference: Group A: Clinic-based structured language rehabilitation; Group B: Parent-led family intervention; and Group C: Specialist-mediated play therapy.

Following the diagnostic assessment, the developmental-behavioral pediatrician held a structured consultation session with parents, during which all three intervention options were presented using standardized informational materials (brochures and video introductions) describing the structure, time commitment, expected benefits, and evidence base for each approach. Parents were informed that all three interventions were established clinical services offered by the department, and that selection was voluntary based on family preference, logistical considerations, and child temperament. No recommendation or prioritization of any specific intervention was made by the physician during the consultation.

Because the study followed an observational case–control design, participants were not randomly assigned to interventions. Instead, treatment selection reflected routine clinical practice and caregiver preference after receiving information about available intervention options.

The study protocol was approved by the Institutional Ethics Committee of People's Hospital of Pingshan (Approval No: 2023-042). Written informed consent was obtained from the parents or legal guardians of all participating children.

### 2.2 Inclusion and exclusion criteria

#### 2.2.1 Inclusion criteria

Children were eligible for inclusion if they met the following criteria: (1) Diagnosis of simple language developmental delay confirmed by a developmental-behavioral pediatrician; (2) Language Developmental Quotient (DQ) between 55 and 75 on the Gesell Developmental Diagnosis Scale, indicating mild delay; (3) Age between 18 and 36 months at enrollment; (4) Residence within the Pingshan district or surrounding areas to allow for consistent follow-up; (5) Parents or guardians able to provide written informed consent and comply with intervention procedures.

#### 2.2.2 Exclusion criteria

Children were excluded if they had: (1) Significant perinatal risk factors (e.g., severe birth asphyxia or grade III–IV intraventricular hemorrhage); (2) Confirmed hearing impairment detected through otoacoustic emission testing and auditory brainstem response assessment; (3) Diagnosed neurological disorders such as epilepsy or cerebral palsy; (4) Autism spectrum disorder or other pervasive developmental disorders based on screening with the Childhood Autism Rating Scale; (5) Structural abnormalities affecting speech production (e.g., cleft palate).

These criteria were applied to reduce potential confounding factors that could independently influence language development outcomes.

### 2.3 Intervention protocols

All interventions were conducted over a 12-month period (Supplementary File S1). Children received interventions according to the management approach selected at baseline.

#### 2.3.1 Note on terminology

The term “specialist” in Group C specifically denotes the active involvement of a developmental-behavioral pediatrician in the design and supervision of interventions. All three interventions were delivered by qualified professionals, and no group is implied to be exclusively “specialized” in nature.

##### 2.3.1.1 Group A: clinic-based structured language rehabilitation

Children in the clinic-based structured rehabilitation group received clinic-based speech and language therapy delivered by certified speech-language pathologists.

Therapy was provided twice weekly, with each session lasting 40 min. Sessions included either individual therapy or small-group therapy (2–3 children) and followed a structured progression focusing on: auditory attention and discrimination; oral–motor and phonological exercises; vocabulary acquisition; phrase and sentence construction; and structured question–answer interaction using visual aids.

These therapy activities were based on commonly used pediatric speech rehabilitation protocols.

##### 2.3.1.2 Group B: parent-Led family intervention

Parents in the family intervention group participated in a 4-week structured training program conducted by a developmental pediatrician and an experienced therapist.

The training curriculum covered: milestones in early language development; responsive communication strategies (e.g., following the child's lead, waiting for responses); designing daily play-based learning activities. After training, parents implemented daily 30-minute home-based intervention sessions targeting four domains: imitation, cognitive engagement, adaptive behavior, and symbolic play.

Parents maintained activity logs documenting session duration, activities performed, and child responses. Logs were reviewed biweekly by clinicians to monitor adherence and provide feedback.

##### 2.3.1.3 Group C: specialist-mediated play therapy

Children in the specialist-mediated play therapy group participated in a structured play-based intervention program designed and supervised by developmental-behavioral pediatricians.

The intervention was delivered by trained speech-language pathologists and early intervention therapists under specialist supervision. Children attended two therapist-facilitated play sessions per week, each lasting 45 min. Sessions were conducted in small groups of approximately 10 children with comparable developmental profiles.

Each session included: an opening ritual to establish routine; two to three structured play activities; a closing storytelling activity.

Therapists used evidence-based communication strategies during play, including parallel talk, narrating the child's actions; expansions, extending child utterances into grammatically complete phrases; communication temptations, encouraging spontaneous communication attempts; and contingent imitation, reinforcing child vocalizations.

Core play activities included: (1) Interactive sensory–motor play, such as action songs with gestures; (2) Symbolic and pretend play, using themed scenarios (e.g., kitchen or medical play); (3) Turn-taking games, designed to promote vocabulary use and communicative exchange; and (4) Collaborative constructive play, such as block building and puzzle solving.

##### 2.3.1.4 Parent involvement

Parents observed one therapy session per week through a one-way observation window. Following observation, clinicians provided 20-minute coaching sessions to help parents implement play-based communication strategies in daily routines.

### 2.4 Intervention fidelity monitoring

To ensure consistency across intervention approaches, fidelity monitoring procedures were implemented.

For Groups A and C, two randomly selected therapy sessions per month were reviewed by an independent senior speech-language pathologist using a standardized fidelity checklist assessing: adherence to session structure, implementation of targeted communication strategies, and therapist responsiveness to child behavior. For Group B, fidelity was assessed through review of parent-completed activity logs documenting home intervention sessions. Average fidelity scores across the study period were: Group A: 92% adherence; Group C: 89% adherence; Group B: 78% adherence (based on reported adherence to daily activities).

### 2.5 Outcome measures

The primary outcome was the change in language developmental ability as measured by the Language Developmental Quotient (DQ) derived from the Gesell Developmental Diagnosis Scale. The DQ was calculated using the formula: DQ = (Developmental Age/Chronological Age) × 100. Interpretation of DQ scores was defined as: 55–75: mild language delay; 76–85: borderline language development; >85: within normal range.

Assessments were conducted at three time points: Baseline (T0), 6 months (T1), and 12 months (T2). All evaluations were performed by a trained developmental assessor who was blinded to intervention group allocation and not involved in intervention delivery.

### 2.6 Statistical analysis

Statistical analyses were conducted using SPSS version 25.0 (IBM Corp.). Continuous variables were presented as mean ± standard deviation, while categorical variables were summarized using frequencies and percentages. Baseline characteristics between groups were compared using One-way analysis of variance (ANOVA) for continuous variables and Chi-square tests for categorical variables. Changes in language DQ over time were evaluated using repeated-measures ANOVA, with time (T0, T1, T2) as the within-subject factor and intervention group as the between-subject factor. Where significant interactions were observed, Bonferroni-adjusted pairwise comparisons were conducted. Effect sizes were reported as partial eta-squared (*η*^2^p). A two-sided *p*-value < 0.05 was considered statistically significant.

## 3 Results

### 3.1 Participant characteristics and baseline data

A total of 180 children with mild language developmental delay were included in the study. Participants were distributed across three intervention groups based on the clinical management strategy selected following physician consultation and parental preference: Clinic-Based Structured Language Rehabilitation (Group A, *n* = 60), Parent-Led Family Intervention (Group B, *n* = 60), and Specialist-Mediated Play Therapy (Group C, *n* = 60). All participants completed the 12-month follow-up period. The high retention rate was achieved through consistent follow-up procedures, including appointment reminders, flexible scheduling, and regular communication with caregivers.

**Figure 1:**
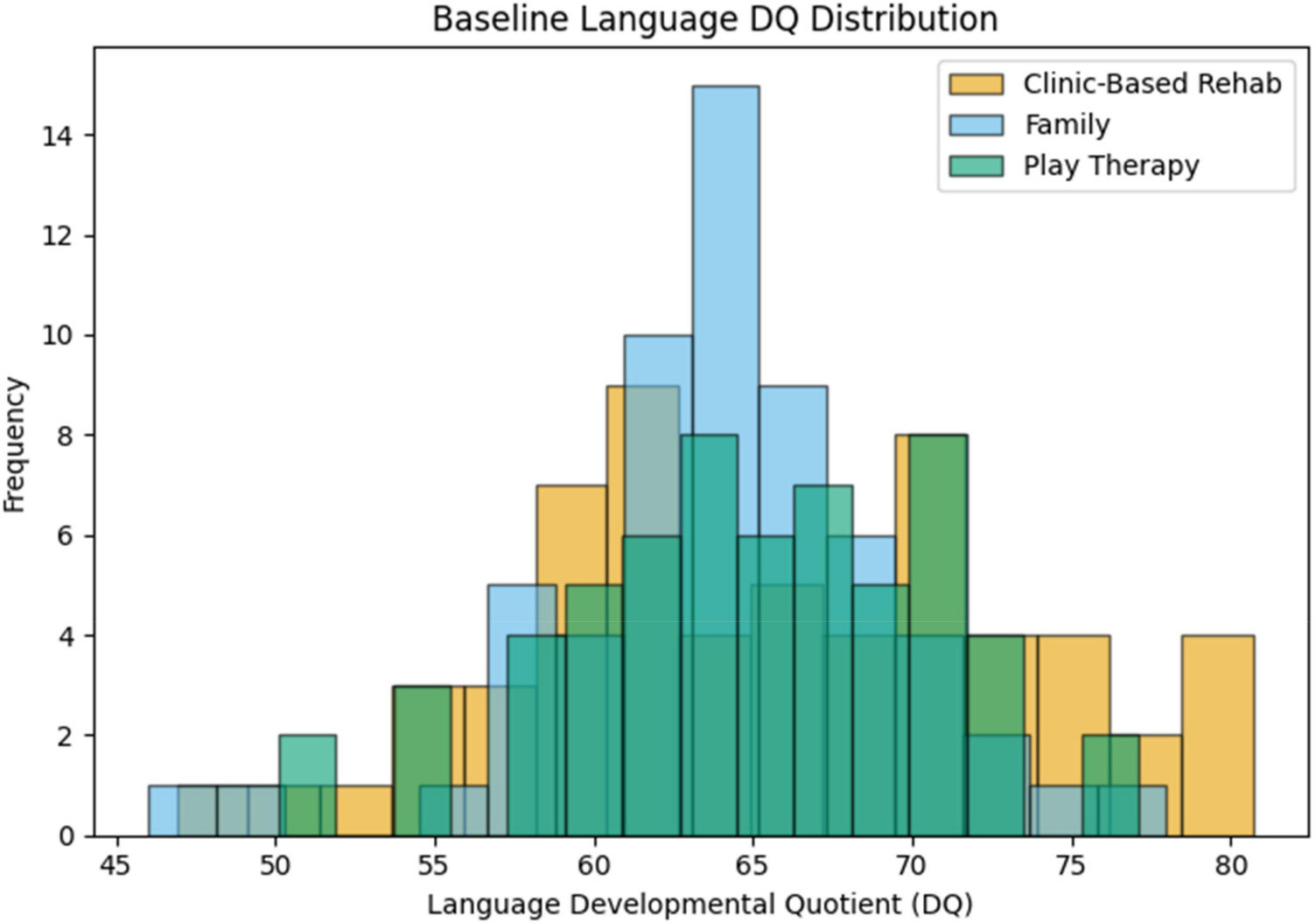
Frequency distribution of language developmental quotient (DQ) scores at baseline for the three intervention groups. Histograms are overlaid with 15 bins; each group is represented by a different color. The similarity of the distributions reflects comparable baseline characteristics among the non-randomized groups.

Baseline demographic and clinical characteristics are summarized in Table 1. The mean age of participants was comparable across groups (Group A: 26.3 ± 5.4 months; Group B: 26.8 ± 5.1 months; Group C: 25.9 ± 5.7 months; *p* = 0.651). Gender distribution was balanced across groups (50% male and 50% female in each group). Mean baseline language Developmental Quotient (DQ) scores were also similar among groups (Group A: 65.1 ± 6.5; Group B: 64.9 ± 5.8; Group C: 65.4 ± 6.2; *p* = 0.892). No statistically significant differences were observed in baseline demographic variables, including maternal education level or initial language severity distribution (all *p* > 0.05), suggesting that the groups were broadly comparable prior to intervention despite non-randomized allocation. The baseline distribution of language DQ scores across the three groups is illustrated in Figure 1, which demonstrates similar distributions of mild language delay at study entry.

**Table 1:** Baseline demographic and clinical characteristics of participants by intervention group.

Characteristic	Group A: Clinic-Based Rehabilitation (*n* = 60)	Group B: Family Intervention (*n* = 60)	Group C: Play Therapy (*n* = 60)	*p*-value
Age, months (Mean ± SD)	26.3 ± 5.4	26.8 ± 5.1	25.9 ± 5.7	0.651
Age Stratum, *n* (%)				0.934
18–24 months	20 (33.3)	19 (31.7)	21 (35.0)	
25–30 months	20 (33.3)	21 (35.0)	19 (31.7)	
31–36 months	20 (33.3)	20 (33.3)	20 (33.3)	
Gender, *n* (%)				1.000
Male	30 (50.0)	30 (50.0)	30 (50.0)	
Female	30 (50.0)	30 (50.0)	30 (50.0)	
Baseline Language DQ (Mean ± SD)	65.1 ± 6.5	64.9 ± 5.8	65.4 ± 6.2	0.892
DQ Severity at Baseline, *n* (%)				1.000
55–65 (Lower Mild)	28 (46.7)	29 (48.3)	27 (45.0)	
66–75 (Upper Mild)	32 (53.3)	31 (51.7)	33 (55.0)	
Maternal Education, *n* (%)				0.781
High School or Below	22 (36.7)	25 (41.7)	24 (40.0)	
College/University	30 (50.0)	28 (46.7)	29 (48.3)	
Postgraduate	8 (13.3)	7 (11.7)	7 (11.7)	

Data are presented as mean ± standard deviation or number (percentage). No statistically significant differences were observed between groups (*p* > 0.05 for all comparisons), indicating balanced baseline characteristics despite non-randomized allocation.

### 3.2 Language developmental quotient outcomes

Changes in language Developmental Quotient (DQ) scores across the three assessment time points are presented in Table 2.

**Table 2:** Language developmental quotient (DQ) across time by intervention group (mean ± SD).

Group	Baseline (T0)	6 Months (T1)	12 Months (T2)
A: Clinic-Based Rehabilitation	65.1 ± 6.5	70.3 ± 7.1	80.5 ± 7.2
B: Family Intervention	64.9 ± 5.8	70.1 ± 6.0	80.0 ± 5.8
C: Play Therapy	65.4 ± 6.2	74.5 ± 6.1^a^^,^^b^	86.3 ± 6.5^a^^,^^b^

a Significantly different from Group A at the same time point (*p* < 0.001).

b Significantly different from Group B at the same time point (*p* < 0.001). Group A: Clinic-based structured rehabilitation; Group B: Family intervention; Group C: Specialist-mediated play therapy.

Repeated-measures analysis of variance demonstrated a significant effect of time on language DQ scores (*F* = 315.45, *p* < 0.001), indicating that language ability improved over the 12-month period across all intervention groups. A significant group effect was also observed (*F* = 4.95, *p* = 0.009), suggesting differences in overall language outcomes among the intervention strategies. Importantly, a significant interaction between time and group was detected (*F* = 32.73, *p* < 0.001), indicating that the trajectory of language development differed among the three intervention groups.

Mean language DQ scores increased progressively over time in all groups. However, children participating in the specialist-mediated play therapy program (Group C) demonstrated greater improvement compared with those in the clinic-based structured rehabilitation (Group A) and parent-led intervention groups (Group B).

At 6 months, the mean DQ in Group C was significantly higher than in Group A (mean difference = 4.2, 95% CI: 2.1–6.3, *p* < 0.001) and Group B (mean difference = 4.4, 95% CI: 2.3–6.5, *p* < 0.001). No significant difference was observed between Groups A and B (*p* = 1.000). At 12 months, the difference between groups remained evident. Group C demonstrated higher mean DQ scores than Group A (mean difference = 5.8, 95% CI: 3.3–8.3, *p* < 0.001) and Group B (mean difference = 6.3, 95% CI: 3.8–8.8, *p* < 0.001). Again, Groups A and B did not differ significantly (*p* = 1.000). Effect size analysis indicated large differences between Group C and the comparison groups at 12 months (Group C vs. Group A: Cohen's d = 0.84; Group C vs. Group B: Cohen's d = 0.98), whereas the difference between Groups A and B was negligible (Cohen's d = 0.08).

### 3.3 Distribution of language development categories

To provide a clinically meaningful interpretation of language outcomes, children were categorized according to DQ ranges representing mild delay (55–75), borderline development (76–85), and normal language function (>85).

**Figure 2:**
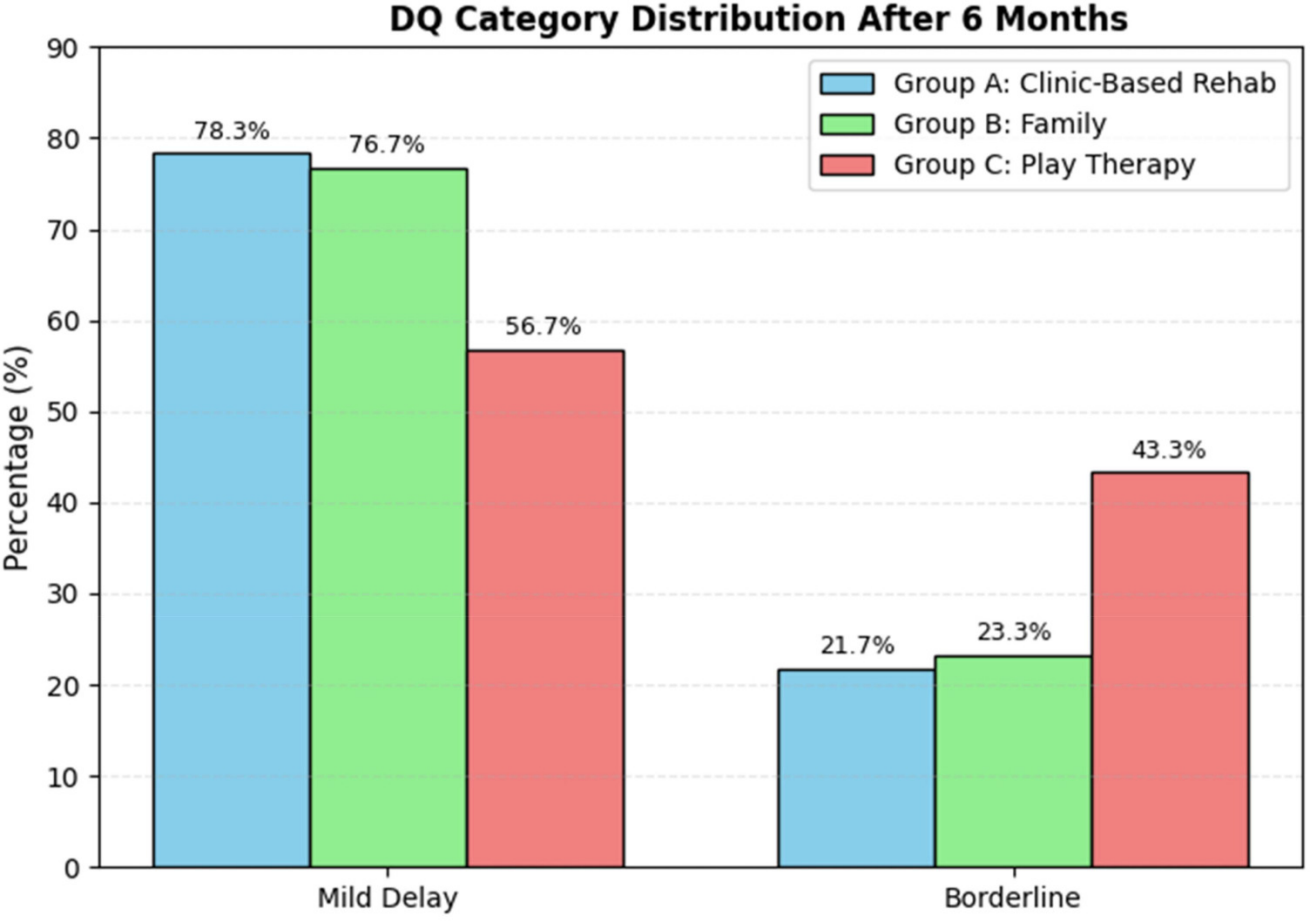
Frequency distribution of language DQ categories after 6 months of intervention. The chart illustrates the shift from Mild Delay to Borderline status (DQ 76–85). Group C shows a substantially larger proportion of children in the Borderline category (43.3%) compared to Group A (21.7%) and Group B (23.3%).

**Figure 3:**
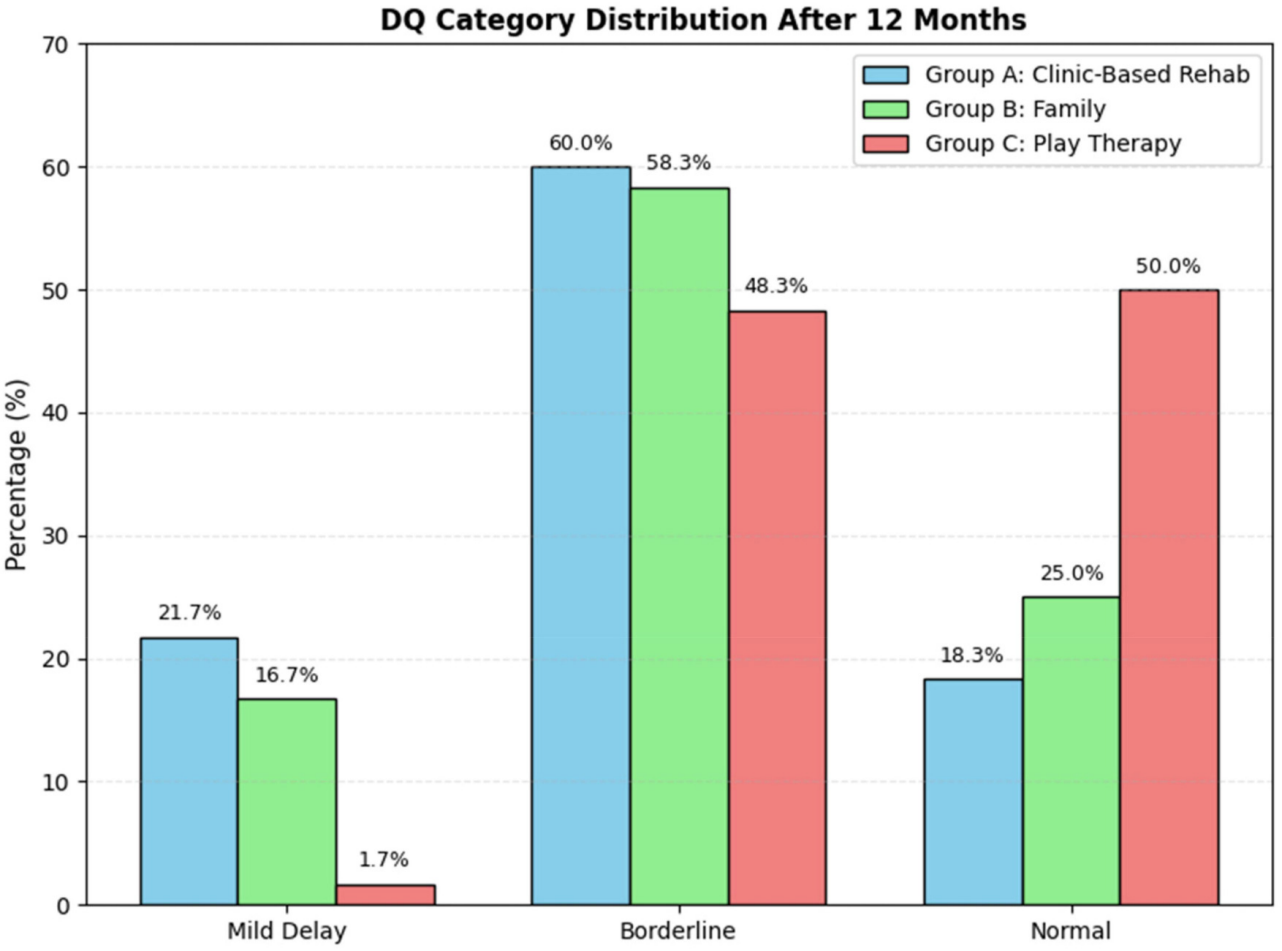
Frequency distribution of language DQ categories after 12 months of intervention. The chart shows the progression into the Normal range (DQ >85). Group C demonstrates a markedly higher proportion of children achieving Normal language status (50.0%) compared to Group A (18.3%) and Group B (25.0%).

**Figure 4:**
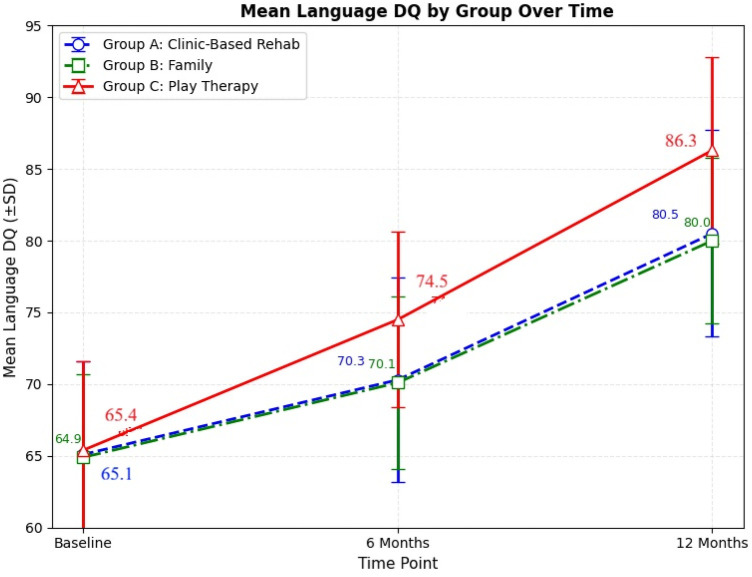
Line chart depicting the mean language developmental quotient (DQ) for each intervention group across the three assessment time points (baseline, 6 months, 12 months). Error bars represent standard deviation. The steeper slope for Group C visualizes the significant Time × Group interaction (*p* < 0.001), suggesting a differential developmental trajectory for the specialist-mediated play therapy approach.

At 6 months, a larger proportion of children in the specialist-mediated play therapy group transitioned from mild delay to the borderline language category. Specifically, 43.3% of children in Group C reached borderline language levels compared with 21.7% in Group A and 23.3% in Group B (Figure 2). At 12 months, improvements were observed across all groups, but the proportion of children achieving normal language development was highest in the specialist-mediated play therapy group. Half of the children in Group C (50.0%) achieved language scores within the normal range, compared with 18.3% in Group A and 25.0% in Group B (Figure 3). These distributions illustrate a greater shift toward typical language development among children receiving the specialist-mediated play intervention. The progression of mean DQ scores across the three time points is illustrated in Figure 4, which shows a steeper improvement trajectory in the specialist-mediated play therapy group.

### 3.4 Exploratory analysis by baseline severity

An exploratory analysis was conducted to evaluate whether baseline language severity influenced intervention outcomes. Participants were categorized into two subgroups based on baseline DQ scores: Lower Mild Delay: DQ 55–65; Upper Mild Delay: DQ 66–75.

A mixed-model analysis did not demonstrate a significant three-way interaction between group, baseline severity, and time (*F* = 1.24, *p* = 0.29). This suggests that the pattern of improvement associated with the specialist-mediated play therapy approach was generally consistent across children with different baseline severity levels within the mild delay range (Table 3). Nevertheless, children in the upper mild delay subgroup exhibited higher absolute DQ scores at all time points across intervention groups. Due to the exploratory nature of this analysis and smaller subgroup sample sizes, these findings should be interpreted cautiously.

**Table 3:** Final DQ (12-months) by intervention group and baseline severity subgroup (mean ± SD).

Baseline Severity	Group A: Clinic-Based Rehabilitation	Group B: Family Intervention	Group C: Play Therapy
Lower Mild (DQ 55–65)	77.8 ± 6.9	77.1 ± 5.5	84.1 ± 7.0
Upper Mild (DQ 66–75)	82.8 ± 6.8	82.5 ± 5.0	88.1 ± 5.8

Lower Mild: baseline DQ 55–65; Upper Mild: baseline DQ 66–75.

### 3.5 Subgroup analysis by Age and gender

Additional analyses examined whether intervention outcomes differed according to age group or gender. No significant interactions were observed between intervention group and age subgroup (18–24 months, 25–30 months, 31–36 months) or gender (all *p* > 0.05). These findings suggest that improvements in language development associated with the interventions were broadly consistent across demographic subgroups. Detailed subgroup data are presented in Supplementary Table S2.

## 4 Discussion

### 4.1 Principal findings

This pilot case–control study evaluated the association between three commonly used early language intervention approaches, including clinic-based structured language rehabilitation, parent-led family intervention, and specialist-mediated play therapy, and changes in language developmental outcomes in children aged 18–36 months with mild language developmental delay.

Across all intervention strategies, children demonstrated significant improvement in language developmental quotient (DQ) scores over the 12-month follow-up period, reflecting the potential benefits of early language intervention in this population. However, the magnitude and trajectory of improvement differed among the intervention approaches. Children participating in the specialist-mediated play therapy program exhibited greater increases in language DQ scores compared with those receiving clinic-based structured rehabilitation or parent-led home interventions. In addition, a higher proportion of children in this group transitioned from mild delay to borderline or normal language developmental ranges during follow-up. Importantly, because the present study followed a non-randomized observational case–control design, these findings should be interpreted as associations rather than evidence of causal treatment effects. Nevertheless, the results provide preliminary insights into how different intervention models may influence language development trajectories in young children with mild delays.

### 4.2 Interpretation of findings

The greater improvement observed among children receiving specialist-mediated play therapy may be related to several characteristics of this intervention approach.

First, play-based therapy provides a naturalistic communication context that encourages spontaneous language use. During interactive play, children are exposed to meaningful linguistic input while actively participating in social exchanges. Previous developmental research has shown that such responsive communication environments facilitate vocabulary acquisition, joint attention, and pragmatic language skills, which are essential components of early language development (6, 15). Second, specialist-mediated play sessions incorporate structured communication strategies, such as parallel talk, expansions, and contingent imitation. These techniques are widely recognized in early intervention frameworks as effective methods for scaffolding children's language production and reinforcing communicative attempts. Third, the play therapy program in the present study involved regular therapist–child interaction combined with guided parental observation and coaching, which may have enhanced the consistency of language stimulation in both clinical and home environments.

It is important to acknowledge that the three intervention models differed not only in therapeutic approach but also in the professional status and intensity of therapist involvement. Group A received clinic-based therapy from certified speech-language pathologists; Group B involved primarily parent-led implementation with initial training by a developmental pediatrician and therapist; and Group C combined trained therapist delivery with developmental-behavioral pediatrician supervision and design. The greater improvement observed in Group C may therefore reflect the combined contribution of play-based methodology, higher professional contact intensity, and specialist pediatrician oversight in tailoring developmental profiles. Future randomized studies should standardize therapist qualifications and contact hours across arms to isolate the specific contribution of the play-based model from professional intensity effects.

Together, these elements may help explain the relatively stronger language gains observed in this group, although further controlled studies are required to confirm this possibility.

### 4.3 Comparison with previous research

The findings of this study are broadly consistent with previous literature emphasizing the importance of interactive and play-based communication interventions in early childhood language delay.

Several studies have demonstrated that naturalistic developmental behavioral interventions, which integrate structured teaching strategies into play routines, can support language development in toddlers with communication delays (9, 16). These interventions emphasize responsive communication, child-led interaction, and contextualized language exposure, principles that were incorporated into the specialist-mediated play therapy model evaluated in this study.

Parent-led interventions have also been widely recommended in early language intervention programs because they promote daily language stimulation within natural family environments (17). However, the effectiveness of home-based interventions often depends on parental training, consistency of implementation, and caregiver confidence in applying therapeutic strategies. Variability in these factors may partly explain the relatively modest improvements observed in the parent-led intervention group in the present study. Similarly, clinic-based structured speech therapy remains a core component of pediatric language rehabilitation (18, 19). While structured therapy sessions can effectively target specific linguistic skills, limited therapy frequency and reduced opportunities for naturalistic communication may influence the overall pace of language acquisition.

Taken together, these findings highlight the potential value of integrating structured therapeutic techniques with play-based and socially interactive learning contexts when designing early language intervention programs.

### 4.4 Clinical implications

Early identification and intervention are widely recognized as critical factors in improving developmental outcomes for children with language delay. The results of this pilot study suggest that structured play-based interventions delivered by trained specialists may represent a promising strategy for supporting language development in young children with mild delays.

From a clinical perspective, play-based therapy offers several potential advantages. The approach is developmentally appropriate for toddlers, promotes engagement and motivation, and allows therapists to incorporate language learning opportunities into enjoyable activities (18, 19). Additionally, the inclusion of parent observation and coaching components may enhance caregiver understanding of language facilitation strategies, thereby extending intervention effects beyond therapy sessions (20).

However, implementation of specialist-mediated play therapy programs may require greater professional resources and trained personnel compared with clinic-based structured rehabilitation models. Therefore, integrating elements of play-based communication strategies into existing early intervention services may represent a feasible approach for improving clinical outcomes.

The present findings also carry implications for the role of developmental-behavioral pediatricians in early language intervention. As highlighted in the Introduction, developmental-behavioral pediatricians possess expertise in neurodevelopmental assessment and individualized developmental profiling that allows for tailored intervention design. In this study, the integration of this diagnostic expertise with structured play-based therapy appeared to enhance developmental outcomes. This suggests that pediatricians may serve not only as diagnosticians but as active architects of intervention strategy, bridging the gap between assessment and therapeutic implementation. Future service models might consider formalizing this “specialist-mediated” role within multidisciplinary early intervention teams, leveraging pediatric developmental expertise to inform individualized play-based programs rather than relying solely on generic rehabilitation protocols.

### 4.5 Study limitations

Several limitations should be considered when interpreting the findings of this study.

First, the absence of randomization represents the most important limitation of this study. Intervention selection was influenced by parental preference and clinical consultation, which may introduce selection bias and confounding by indication. Consequently, these findings demonstrate association rather than causation and should be interpreted with appropriate caution.

Second, the single-center design and relatively modest sample size (*n* = 180) limit both generalizability to broader populations and statistical power to detect smaller effect sizes or robustly explore subgroup interactions. The exploratory subgroup analyses by baseline severity, age, and gender should be interpreted cautiously, given the reduced power in stratified comparisons. Multicenter collaborations with larger cohorts are needed to validate these preliminary findings across diverse clinical and demographic settings.

Third, the study relied on a single primary outcome measure (language DQ from the Gesell Developmental Diagnosis Scale). Although widely used and standardized for Chinese pediatric populations, additional measures, such as expressive vocabulary size, receptive language ability, pragmatic language skills, or parent-reported communication functional outcomes, would provide a more comprehensive and nuanced evaluation of intervention effects. Future studies should employ a multi-dimensional language and communication battery to capture the breadth of potential treatment impacts.

Fourth, adherence to the parent-led intervention program was monitored through self-reported activity logs, which may be subject to reporting bias. The use of standardized fidelity checklists and independent session review for Groups A and C represents a methodological strength that enhances the internal validity of our intervention comparisons; however, similar objective monitoring was not feasible for Group B. Finally, the follow-up period of 12 months, although sufficient to observe early language changes, does not allow evaluation of long-term developmental outcomes, such as later academic performance or social communication skills. Future studies should address these limitations through larger multicenter designs, randomized controlled methodologies, and longer follow-up periods.

### 4.6 Future research directions

The results of this pilot study highlight several directions for future research.

Large-scale, multicenter randomized controlled trials with adequate power, standardized intervention protocols, blinded outcome assessment, and long-term follow-up are essential to confirm the causal efficacy of specialist-mediated play therapy, elucidate underlying neurodevelopmental mechanisms, and optimize intervention dosage, intensity, and delivery formats for diverse pediatric populations. Such studies should incorporate standardized language outcome measures, detailed intervention fidelity monitoring, and longer-term follow-up assessments. Future research should also examine how parent training intensity, therapist involvement, and intervention frequency influence treatment outcomes.

Additionally, hybrid intervention models warrant investigation. Combining the structured play-based approach evaluated in this study with intensive parent training components and digital health tools (e.g., telehealth-based parent coaching, mobile application-guided home practice, or wearable device monitoring of language exposure) may enhance intervention accessibility, consistency, and scalability. Such hybrid models could potentially deliver the benefits of specialist-mediated design while reducing the resource intensity that limits widespread implementation, particularly in resource-limited or rural settings.

Finally, investigating the neurodevelopmental mechanisms underlying language improvement, including changes in social attention and cognitive engagement during play-based interactions, may provide further insights into optimizing early intervention strategies.

## 5 Conclusion

This pilot case-control study examined the association between three early intervention strategies, clinic-based structured language rehabilitation, parent-led family intervention, and specialist-mediated play therapy, and language developmental outcomes in children aged 18–36 months with mild language developmental delay. All intervention approaches were associated with improvements in language developmental quotient scores over the 12-month follow-up period, highlighting the potential benefits of early intervention in supporting language acquisition during early childhood. Among the intervention models evaluated, children participating in the specialist-mediated play therapy program demonstrated greater gains in language developmental scores and a higher likelihood of achieving age-appropriate language levels compared with the other intervention groups. Given the non-randomized observational design and pilot nature of this study, these findings should be interpreted cautiously as associations rather than causal effects. The results suggest that structured play-based interventions delivered by trained specialists may represent a promising approach for supporting language development in young children with mild delay. Further large-scale, multicenter randomized controlled trials are needed to confirm these findings, explore underlying mechanisms, and determine the most effective and scalable early intervention strategies for children with language developmental delay.

## Data Availability

The original contributions presented in the study are included in the article/Supplementary Material, further inquiries can be directed to the corresponding author/s.
