# Supplementary material for: Specialist-mediated play therapy for toddlers with language developmental delay: a single-center observational pilot study
Source: Front Rehabil Sci. 2026 May 29;7:1830893. doi: 10.3389/fresc.2026.1830893 (PMC13260146; doi:10.3389/fresc.2026.1830893)
Supplement: Supplementary file 1 [file Datasheet1.pdf]

Supplementary Materials

Supplementary Table S1. Summary of Intervention Components and Estimated Professional Contact Hours

**Component**	**Group A: Therapist-Directed**	**Group B: Parent-Led Family Intervention**	**Group C: Specialist-Mediated Play Therapy**
Direct child therapy sessions	2×/week × 40 min × 48 weeks = ~64 hours	None	2×/week × 45 min × 48 weeks = ~72 hours
Parent training/coaching	None	4-week workshop (~16 hours) + bi-weekly logbook review (~30 min × 24 = ~12 hours) = ~28 hours total	Weekly 20-min coaching × 48 weeks = ~16 hours
Total professional contact	~64 hours	~28 hours	~88 hours
Home practice	None required	Daily 30-min sessions (parent-led)	Encouraged with coached strategies
Specialist involvement	Speech-language pathologist	Developmental pediatrician (training only)	Pediatrician design/supervision + therapist delivery

Note: Professional contact hours are estimates based on protocol specifications. Group A was delivered by certified speech-language pathologists; Group B by parents following initial training by a developmental pediatrician and experienced therapist; Group C by trained speech-language pathologists and early intervention therapists under developmental-behavioral pediatrician supervision. All three interventions were delivered by qualified professionals.

Supplementary Table S2. Detailed Language DQ Scores by Age and Gender Subgroups (Mean ± SD)

Group	Gender	Age Group	Baseline (T0)	6 Months (T1)	12 Months (T2)
Group A (Clinic-Based Structured Rehabilitation)	Male	18-24 months	65.86 ± 7.13	70.86 ± 8.38	81.14 ± 6.91
		25-30 months	64.71 ± 5.82	71.29 ± 6.63	80.43 ± 6.11
		31-36 months	62.33 ± 6.51	66.33 ± 7.10	75.67 ± 10.26
	Female	18-24 months	68.20 ± 7.98	73.40 ± 8.33	82.80 ± 6.87
		25-30 months	65.89 ± 6.11	72.56 ± 5.81	79.89 ± 7.03
		31-36 months	64.56 ± 6.46	69.44 ± 6.56	80.22 ± 6.67
Group B (Family Intervention)	Male	18-24 months	68.43 ± 4.47	72.00 ± 4.47	83.86 ± 5.73
		25-30 months	65.00 ± 5.66	69.71 ± 7.39	78.00 ± 5.00
		31-36 months	64.67 ± 7.66	68.67 ± 7.06	79.00 ± 7.21
	Female	18-24 months	66.17 ± 5.38	70.50 ± 5.58	79.00 ± 5.55

Group	Gender	Age Group	Baseline (T0)	6 Months (T1)	12 Months (T2)
		25-30 months	64.00 ± 5.20	69.29 ± 4.96	78.43 ± 5.50
		31-36 months	64.57 ± 5.97	70.14 ± 6.31	79.29 ± 5.12
	Male	18-24 months	66.25 ± 6.59	73.75 ± 6.56	85.63 ± 6.16
		25-30 months	65.00 ± 8.40	72.60 ± 8.30	85.60 ± 8.11
		31-36 months	68.67 ± 5.39	77.00 ± 4.98	88.17 ± 5.91
Group C (Play Therapy)	Female	18-24 months	68.13 ± 6.13	76.38 ± 6.59	89.38 ± 6.46
		25-30 months	66.71 ± 4.82	75.14 ± 4.18	87.29 ± 5.50
		31-36 months	63.33 ± 7.58	71.67 ± 7.17	82.83 ± 7.86

Notes: Age Group 1: 18–24 months; Age Group 2: 25–30 months; Age Group 3: 31–36 months. This detailed breakdown shows the consistency of the intervention effect across different demographic subgroups.
